# The association between generative AI use and university students’ critical thinking: a moderated mediation model of GenAI feedback literacy and reflective thinking

**DOI:** 10.3389/fpsyg.2026.1939286

**Published:** 2026-09-22

**Authors:** Zhen Yan, Zhuotao Fang, Weixing Zou

**Affiliations:** Minzu Normal University of Xingyi, Xingyi, China

**Keywords:** critical thinking, feedback literacy, information literacy, post-secondary education, reflective thinking

## Abstract

Generative artificial intelligence (GenAI) has rapidly become an everyday learning resource for university students, yet evidence on whether such use is associated with stronger or weaker critical thinking is sharply divided. Recent work documents both cognitive scaffolding and “metacognitive laziness” effects, suggesting that the AI-cognition relationship is contingent on how students engage with AI-generated feedback. Drawing on the extended-mind framework, cognitive load theory and feedback-literacy theory, we test a cross-sectional moderated mediation model in which GenAI feedback literacy mediates the association between GenAI use and critical thinking, operationalised as self-reported critical analytical skills, and reflective thinking moderates both the direct path and the path from feedback literacy to critical thinking. A sample of 421 Chinese undergraduates (69.6% female) completed validated self-report measures, and partial least squares structural equation modelling (PLS-SEM, 5,000 bootstrap resamples) was used to estimate the model. GenAI use significantly predicted critical thinking (*β* = 0.257, *p* < 0.001) and feedback literacy (*β* = 0.438, *p* < 0.001), and feedback literacy in turn predicted critical thinking (*β* = 0.421, *p* < 0.001). The indirect effect through feedback literacy was substantial (*β* = 0.185, 95% CI [0.134, 0.254]; VAF = 71.98%), and the direct effect dropped to non-significance once the mediator entered the model, a pattern consistent with near-complete mediation. Reflective thinking moderated the structure of the relationship: at low reflective thinking, GenAI use was directly associated with critical thinking; at high reflective thinking, the association was carried almost entirely by feedback literacy, and the conditional indirect effect was strongest. Because all variables were measured concurrently by self-report, the findings describe associations consistent with, but not demonstrative of, the proposed mechanism. They are nonetheless consistent with a view of GenAI use as a context-sensitive cognitive resource: its association with self-reported critical analytical skills depends on whether students can interpret, judge and act on AI-generated feedback, and whether they bring a reflective stance to the interaction. Implications for AI-pedagogy integration, feedback-literacy curricula and reflective course design in higher education are discussed.

## 1 Introduction

The diffusion of generative artificial intelligence (GenAI) tools such as ChatGPT, Claude, Gemini and Wenxin Yiyan has reconfigured the everyday learning practises of university students worldwide. Within 2 years of the public release of large language models, AI assistants have become routine sources of explanation, drafting, summarisation and feedback in academic life (Yan et al., 2024; Zhao et al., 2025). Recent surveys indicate that a majority of undergraduates now consult GenAI for at least one course task per week, with usage rates still rising sharply (Ravselj et al., 2025). For higher-education researchers and educators, the central question has therefore shifted from whether students will use GenAI to how its use relates to the cognitive capacities that universities are expected to develop, foremost amongst them critical thinking.

Critical thinking, broadly conceived as the disciplined ability to analyse evidence, evaluate arguments and reflect on the warrant of one’s conclusions (Facione, 2011), is widely treated as a signature graduate attribute and a core indicator of the value added by higher education (Dinsmore and Fryer, 2023). Yet the empirical literature on GenAI’s contribution to critical thinking is strikingly inconsistent. One body of work warns that frequent reliance on GenAI promotes cognitive offloading and “metacognitive laziness”: when learners can outsource analysis to a fluent machine, they invest less effort in independent reasoning, monitor their own thinking less, and ultimately produce shallower work (Fan et al., 2025; Gerlich, 2025; Risko and Gilbert, 2016; Tian and Zhang, 2025; Zhai et al., 2024). A complementary body, however, suggests that GenAI can scaffold higher-order thinking by providing real-time feedback, multi-path problem solving and prompts for self-explanation (Balart et al., 2026; Hwang et al., 2025; Nasr et al., 2025; Wu et al., 2026; Zhao et al., 2025). Two recent systematic reviews of more than 60 empirical studies each conclude that the divergence is not artefactual but is driven by how AI is integrated into learning and how learners engage with its outputs (Balart et al., 2026; Vargas-Murillo et al., 2026). These mixed findings are unlikely to be reconciled by simply estimating an average effect; they point instead to unexamined mechanisms and boundary conditions that determine when GenAI use translates into deeper cognition.

Two such mechanisms motivate the present study. The first is feedback literacy. Carless and Boud’s (2018) framework defines feedback literacy as the capacity to understand, judge and act on feedback information, and a growing literature shows that this capacity is what converts feedback exposure into learning gains (Molloy et al., 2020; Nieminen and Carless, 2023). GenAI interaction is, by design, an extended feedback exchange: prompts elicit responses that learners must interpret, evaluate and refine across multiple turns (Yan et al., 2024; Zhang et al., 2026). A wave of recent work has therefore proposed AI-specific feedback-literacy constructs and shown that students with stronger AI feedback literacy take up AI-generated feedback more productively (Jin et al., 2025; Liu and Deris, 2025; Zhan and Yan, 2026; Zhan et al., 2025). Whether GenAI feedback literacy accounts for the association between GenAI use and critical thinking, however, has not been tested in a well-specified mediation model.

The second mechanism is reflective thinking. The same GenAI exchange can be processed superficially or deeply, depending on the learner’s habitual disposition to monitor and revise their own reasoning (Kember et al., 2000; Merkebu et al., 2024). Highly reflective learners tend to treat AI-generated content as material for inspection and comparison, whereas less reflective learners are more likely to accept it as authoritative and proceed (Chan and Lee, 2021; Walton et al., 2026; Xiao and Liu, 2025). Fan et al.’s (2025) randomised study showed that even when motivation is held constant, AI-supported learners exhibit fewer self-regulatory and metacognitive moves than learners with human expert support, suggesting that the cognitive return from GenAI use depends on the metacognitive stance the learner brings. If this is correct, reflective thinking should function as a boundary condition that alters the structure of the association between GenAI use and critical thinking: at high reflectivity the association should be carried by feedback literacy, whilst at low reflectivity it should remain direct and unmediated. To our knowledge, no study has yet modelled the joint operation of GenAI feedback literacy and reflective thinking on critical thinking.

The present study addresses this gap by testing a moderated mediation model amongst 421 Chinese undergraduates. We ask three research questions:

*RQ1*. Does GenAI use predict university students’ critical thinking, directly and through GenAI feedback literacy?

*RQ2*. Does reflective thinking moderate the direct path from GenAI use to critical thinking and the path from GenAI feedback literacy to critical thinking?

*RQ3*. Is the indirect effect of GenAI use on critical thinking via GenAI feedback literacy itself conditional on reflective thinking?

Answering these questions makes three contributions to the educational technology literature. Theoretically, we integrate the extended-mind, cognitive-load and feedback-literacy frameworks to articulate a process model of how GenAI use may relate to higher-order cognition, moving the debate beyond whether AI helps or hurts critical thinking towards the conditions under which each pattern emerges. Empirically, we provide the first test of a moderated mediation model that simultaneously specifies feedback literacy as a mechanism and reflective thinking as a boundary condition, using a large sample and bootstrap-validated PLS-SEM estimation. Practically, the results inform how universities can structure GenAI integration so that increased AI use is more likely to support, rather than undermine, students’ analytical capacity. The remainder of the paper develops the theoretical model and hypotheses (Section 2), describes the methods (Section 3), reports the results (Section 4), and discusses implications, limitations and directions for further research (Sections 5 and 6).

## 2 Theoretical background and hypothesis development

### 2.1 GenAI use and critical thinking

The literature offers no settled answer on whether GenAI use enhances or undermines critical thinking, and the disagreement appears to reflect substantive theoretical differences rather than measurement noise. On one side, scholars argue that the immediacy and high availability of GenAI invite automation bias and cognitive offloading: tasks that once required independent analysis can be transferred to an external system, reducing the depth at which information is processed and evaluated (Risko and Gilbert, 2016; Zhai et al., 2024). Recent randomised evidence sharpens the concern. Fan et al. (2025) found that university students supported by ChatGPT performed comparably to peers supported by a human expert on essay quality, but exhibited markedly fewer self-regulatory and metacognitive processes during the task, a pattern the authors term “metacognitive laziness.” Cross-sectional studies converge on a similar picture: heavier dependence on AI tools is associated with shallower information-processing patterns and weaker analytical engagement (Gerlich, 2025; Tian and Zhang, 2025; Zhai et al., 2024). On the other side, a growing body of work shows that GenAI does not simply replace human cognition; through multi-turn feedback, perspective generation and option comparison, it can scaffold more complex cognitive processing and produce small-to-medium positive effects on higher-order thinking (Vargas-Murillo et al., 2026; Wu et al., 2026; Zhao et al., 2025). When students are asked to question, compare and revise GenAI outputs, real-time feedback and dynamic cognitive support can activate analysis and evaluation processes (Hwang et al., 2025; Nasr et al., 2025; Raitskaya and Tikhonova, 2025; Zhao et al., 2025).

The extended-mind perspective offers a productive way to integrate these views. Cognitive activity is not confined to the brain; it can be distributed across the body and the external environment, with external tools functionally equivalent to internal cognitive processes under specific conditions (Clark, 2008; Clark and Chalmers, 1998). Cognition, on this view, is the interaction between agent and environment, and external resources can substantively shape cognitive activity by participating in information processing. In learning contexts, GenAI does more than supply information: by clarifying questions, unfolding lines of argument, juxtaposing perspectives and revising on demand, it can become a functional component of the learner’s cognitive system (Zhu and Yang, 2026). The crucial qualification is how the tool is used. When deployed as a complementary cognitive partner, learners retain an active cognitive position and engage in continual comparison, questioning and revision, supporting higher-level processing; when deployed as a substitute, the same tool can absorb the cognitive work that learning is meant to elicit (Nasr et al., 2025).

Cognitive load theory provides a complementary mechanistic account. Working-memory resources are finite, and learning tasks involving simultaneous information search, integration and structural organisation generate high extraneous load, crowding out the resources needed for deep processing (Evans et al., 2024; Sweller, 2024). University students typically face precisely this kind of multi-source task environment, and the resulting resource competition limits their investment in analysis, evaluation and inference (Hernandez Sibo et al., 2024; Walraven et al., 2008). The instant generation and iterative feedback features of GenAI can absorb a portion of these lower-level processing tasks, freeing cognitive resources for evidence comparison, argument testing and viewpoint evaluation (Balart et al., 2026). Multi-turn interaction further pushes learners to revise and reconstruct information, producing an analysis-evaluation-revision loop (Celik et al., 2024) that is itself an important manifestation of critical thinking. Empirical work supports this pattern at the aggregate level: across multiple recent studies, GenAI use is positively associated with university students’ critical thinking, and the association operates partly through active inquiry, meaning construction and critical questioning of AI outputs (Balart et al., 2026; Tang et al., 2025; Vargas-Murillo et al., 2026). On these grounds we propose:

*H1*. GenAI use is positively associated with university students’ critical thinking.

### 2.2 The mediating role of GenAI feedback literacy

Although GenAI offers learners abundant information and diverse perspectives, whether such exposure translates into higher-order cognitive capacity hinges on how learners process and use what they receive. A consistent finding across recent reviews is that technology does not directly determine learning outcomes; its effects are channelled through the cognitive processing it elicits (Yan et al., 2024). Feedback literacy provides a powerful theoretical lens for unpacking this channel. Defined as the capacity to understand, evaluate and act on feedback (Carless and Boud, 2018), feedback literacy is the central mechanism by which external information becomes internal cognitive development (Molloy et al., 2020; Nieminen and Carless, 2023). A defining feature of GenAI is its feedback-driven interaction mode (Yan et al., 2024). Unlike traditional information tools, GenAI does not deliver a one-shot answer; through multi-turn dialogue it generates, revises and refines outputs (Balart et al., 2026; Zhang et al., 2026), forming an ongoing feedback loop. GenAI use therefore does more than expand information access: it intensifies the frequency and depth of learners’ feedback engagement (Jin et al., 2025; Zhan and Yan, 2026).

From a feedback-literacy perspective, learning is not the passive reception of information but a continuous regulatory process organised around feedback. Carless and Boud (2018) emphasise that feedback literacy comprises not only comprehension of feedback information but also recognition of its value, judgement of its quality and the capacity to take action on it. Within this framework, GenAI provides an unusually high-frequency interactional environment for feedback-literacy development through several cognitive routes. First, frequent interaction gives learners repeated exposure to feedback, accumulating processing experience and gradually improving their ability to interpret feedback meaning (Zhan and Yan, 2026). Second, optimising prompts to elicit higher-quality outputs trains learners’ understanding of feedback structure and feedback-generation logic, and makes prompt engineering itself a cognitive practise (Knoth et al., 2024; Liu and Deris, 2025). Third, because GenAI outputs lack stable epistemic authority and must be assessed in context, learners are obliged to evaluate relevance and accuracy, which strengthens their capacity to judge feedback quality (Carless and Boud, 2018; Walton et al., 2026). Fourth, as multi-turn interaction proceeds, feedback is converted into specific adjustments, with learners revising prior cognitions and task strategies and thereby reinforcing their ability to act on feedback (Zhan et al., 2025). On these grounds:

*H2*. GenAI use is positively associated with university students’ GenAI feedback literacy.

Critical thinking is commonly defined as the capacity to analyse, evaluate and judge information, centring on examining evidence, parsing argument structure and reflecting on the soundness of conclusions (Facione, 2011). From a cognitive-processing standpoint, critical thinking is not about acquiring information per se but about the deep processing and cognitive reconstruction of what is acquired (Dinsmore and Fryer, 2023). Feedback literacy and critical thinking are highly congruent at the cognitive-structure level. Comprehending feedback requires identifying core content, implicit assumptions and logical relations (Carless and Boud, 2018), processes that closely parallel the analytical component of critical thinking. Evaluating feedback involves judging the credibility and reasonableness of feedback information, foregrounding the examination of evidence quality and inferential validity at the heart of critical thinking (Boud and Molloy, 2013; Zeng and Ravindran, 2025). Acting on feedback involves reconstructing existing cognitive structures or behavioural strategies on the basis of reflection (Carless and Boud, 2018; Molloy et al., 2020). The progressive comprehension–evaluation–action mechanism of feedback literacy thus mirrors the analysis–evaluation–inference cycle of critical thinking, making feedback literacy a plausible cognitive route to critical-thinking development.

In GenAI-supported settings, this congruence becomes consequential. Unlike mere exposure to information, feedback literacy emphasises learners’ active processing and regulation, allowing them to engage in purposeful analysis and judgement in complex information environments (Nieminen and Carless, 2023). Learners with high feedback literacy can identify differences and conflicts amongst AI outputs, integrate them in line with task goals, and form more coherent cognitive representations (Carless and Boud, 2018; Gong et al., 2025). Learners with limited feedback literacy struggle to integrate information effectively; their processing remains shallow, constraining critical-thinking development (Tepgec et al., 2025). Knowledge formation depends on the ongoing processing and integration of external information rather than simple reception (Bransford et al., 2000), and feedback literacy determines whether learners can use information effectively to optimise and reorganise cognitive structures. Accordingly:

*H3*. GenAI feedback literacy is positively associated with university students’ critical thinking.

Taken together, these arguments imply an indirect rather than a direct relationship between GenAI use and critical thinking. Critical thinking does not arise from the volume of information acquired but from whether information is deeply analysed, evaluated and reconstructed (Mayer, 2024). Where learners passively accept GenAI outputs, processing remains shallow and AI engagement is unlikely to translate into critical thinking (Fan et al., 2025; Gerlich, 2025). Conversely, where learners engage actively with feedback—interpreting outputs, identifying core claims and implicit assumptions, judging plausibility through comparison—feedback-centred processing fuels revision and reconstruction (Marton and Säljö, 1976; Risko and Gilbert, 2016). Higher-feedback-literacy learners treat GenAI outputs as testable and revisable; lower-feedback-literacy learners treat them as authoritative and accept them, suppressing deep processing (Carless and Boud, 2018; Walton et al., 2026). The link between GenAI use and critical thinking is therefore not a simple direct relationship but an indirect one mediated by feedback processing. Hence:

*H4*. GenAI feedback literacy mediates the relationship between GenAI use and university students’ critical thinking.

### 2.3 The moderating role of reflective thinking

In GenAI-supported learning, reflective thinking is a strong candidate for the boundary condition that determines the depth of cognitive processing. Although GenAI offers rich information and diverse perspectives, evidence remains divided on whether such use cultivates higher-order cognition (Balart et al., 2026; Fan et al., 2025; Vargas-Murillo et al., 2026). One reading of the divergence is that GenAI’s cognitive consequences depend on the metacognitive stance learners bring to the interaction. Reflective thinking extends beyond comprehension to ongoing scrutiny of one’s reasoning and the monitoring and regulation of cognitive strategies (Kember et al., 2000; Merkebu et al., 2024; Zimmerman, 2002). Highly reflective learners tend to question information sources, compare alternative views and revise existing structures (Chan and Lee, 2021); in GenAI interaction they treat outputs as material to be tested rather than answers to be accepted (Walton et al., 2026; Xiao and Liu, 2025). Less reflective learners are more apt to take GenAI outputs as authoritative, suppress questioning and remain at the surface (Tian and Zhang, 2025), echoing the metacognitive-laziness pattern documented experimentally by Fan et al. (2025).

Reflective thinking is therefore likely to alter the structure of the relationship between GenAI use and critical thinking. At low reflective thinking, GenAI use may show a small, direct association with critical thinking that does not pass through systematic feedback engagement, consistent with the surface-level scaffolding that even passive use can provide. At high reflective thinking, learners pursue cognitive development through deeper feedback-driven processing pathways, attenuating the residual direct effect once the indirect path is in the model. We refer to this contrast between a predominantly direct association at low reflectivity and a predominantly feedback-mediated association at high reflectivity as a dual-route pattern. Hence:

*H5a*. Reflective thinking moderates the relationship between GenAI use and critical thinking, such that the direct effect is stronger at lower levels of reflective thinking.

If feedback literacy is the mechanism that links GenAI use to critical thinking, the same boundary condition should apply to that link. Possessing a capacity does not guarantee that it is converted into a cognitive outcome; whether learners actually engage in comprehension, evaluation and use of relevant information depends on their cognitive disposition (Meyer et al., 2025). Even with strong feedback comprehension and evaluation, learners may or may not push their processing into deeper cognitive territory. Feedback literacy emphasises understanding, judgement and use of feedback, but processing can occur at different cognitive depths. Learners sometimes make surface revisions, accepting suggestions or correcting obvious errors without examining underlying assumptions and argumentative structure (Jensen et al., 2021); in this case, feedback literacy is formally present but its contribution to higher-order cognition remains limited. When learners reflect critically on feedback and reconstruct prior cognitive frameworks, by contrast, feedback becomes a driver of deep learning and cognitive development (Carless and Boud, 2018; Gong et al., 2025). Reflective thinking is the lever in this conversion. Highly reflective learners process feedback at multiple levels—examining the logic behind it, comparing alternatives and reconstructing existing structures (Chan and Lee, 2021)—and so amplify the influence of feedback literacy on critical thinking. Less reflective learners may understand feedback yet apply it mechanically, narrowing the cognitive payoff (Gong et al., 2025; Tepgec et al., 2025). Accordingly:

*H5b*. Reflective thinking moderates the relationship between GenAI feedback literacy and critical thinking, such that the effect is stronger at higher levels of reflective thinking.

Combining H4 and H5b implies a moderated mediation structure: the indirect effect of GenAI use on critical thinking through feedback literacy itself depends on reflective thinking. At low reflective thinking, feedback literacy cannot fully convert AI exposure into critical-thinking gains, so the indirect path is weak; at high reflective thinking, feedback literacy is fully mobilised and the indirect path strengthens. We therefore propose:

*H6*. Reflective thinking moderates the indirect effect of GenAI use on critical thinking via GenAI feedback literacy, with a stronger indirect effect at higher levels of reflective thinking.

### 2.4 The proposed research model

Figure 1 summarises the hypothesised model. GenAI use is posited to be associated with critical thinking overall (H1, the total effect) and indirectly through GenAI feedback literacy (H2, H3, H4). Reflective thinking is modelled as a moderator of the direct path from GenAI use to critical thinking (H5a) and of the path from GenAI feedback literacy to critical thinking (H5b), with the indirect effect itself conditional on reflective thinking (H6). The model integrates an extended-mind reading of GenAI as a complementary cognitive partner with a feedback-literacy account of how AI exposure may relate to cognitive development, and uses reflective thinking to specify when each pathway dominates.

**Figure 1:**
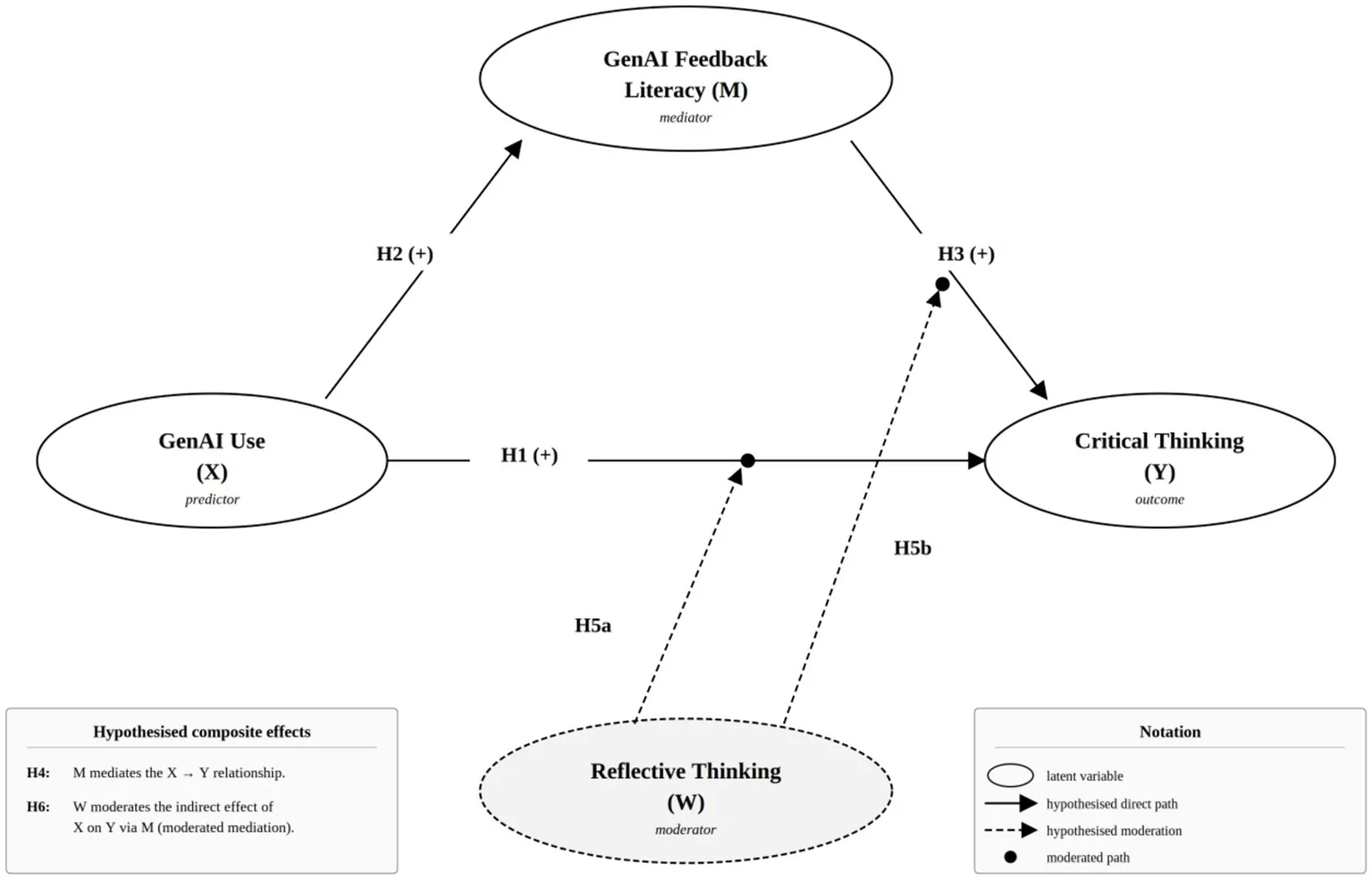
The hypothesised moderated mediation model. Ellipses denote latent variables. Solid arrows represent hypothesised direct relationships; dashed arrows represent hypothesised moderation. Filled dots mark the paths on which reflective thinking is hypothesised to operate. H4 specifies that GenAI feedback literacy mediates the relationship between GenAI use and critical thinking; H6 specifies that this indirect effect is itself conditional on reflective thinking.

## 3 Methods

### 3.1 Participants and procedure

The study used a cross-sectional survey design. Convenience sampling was used to administer paper-and-pencil questionnaires to undergraduate students at universities in mainland China during the 2024–2025 academic year. After data screening for completeness and inattentive responding, 421 valid questionnaires were retained for analysis. Of these participants, 128 (30.4%) identified as male and 293 (69.6%) as female. The sample comprised 116 first-year (27.6%), 101 second-year (24.0%), 164 third-year (39.0%) and 40 fourth-year (9.5%) students, drawn from a range of disciplines. The sample size comfortably exceeds the minimum required for PLS-SEM with the present model complexity, satisfying both Cohen’s power conventions and the inverse-square-root rule (Hair et al., 2021; Kock and Hadaya, 2018).

All participation was voluntary and anonymous. Before completing the questionnaire, students were informed of the study purpose and the protections in place; they gave written informed consent and were assured that responses would be used solely for academic research and that they could withdraw at any time. The study was conducted in accordance with the ethical guidelines of the authors’ institution and approved by the relevant Institutional Review Board. To reduce potential common method bias, the questionnaire was pilot-tested on 30 undergraduates not included in the final sample, items were arranged so that the predictor and criterion measures were separated, and procedural remedies recommended by Podsakoff et al. (2003) were applied (e.g., guaranteed anonymity, balanced item polarity, and clear instructions emphasising that there were no right or wrong answers).

### 3.2 Measures

All scales were administered in Chinese; English-language scales were translated and back-translated by two bilingual researchers, with discrepancies resolved by a third reviewer and the wording revised after a small-scale pilot. Items were rated on a five-point Likert-type scale (1 = strongly disagree, 5 = strongly agree) unless otherwise noted. Scale scores reported in Table 1 were calculated as the sum of the constituent items, with higher scores indicating higher levels of the construct; for the PLS-SEM measurement model, dimension and parcel scores were computed as item means (Section 3.3), which is immaterial for estimation because PLS-SEM standardises all indicators.

**Table 1:** Means, standard deviations and zero-order correlations.

Variable	M ± SD	1	2	3	4
1. GenAI use	56.55 ± 9.44	1			
2. GenAI feedback literacy	130.95 ± 18.37	0.451***	1		
3. Critical thinking	37.60 ± 5.29	0.244***	0.440***	1	
4. Reflective thinking	56.42 ± 7.44	0.262***	0.510***	0.591***	1

*N* = 421. ***p < 0.001.

#### 3.2.1 GenAI use

GenAI use was measured with the 17-item College Students’ Generative AI Use Survey developed by Li et al. (2024). The instrument captures students’ use of GenAI across four typical scenarios identified through literature review and semi-structured interviews with undergraduates: course learning, research activities, daily life, and further-study and job seeking. Sample items include “I use generative AI to answer the questions my teachers ask in class”, “I use generative AI to assist me in selecting research questions”, “When I encounter difficulties in life (such as diet, financial management or social relationships), I turn to generative AI for help”, and “I ask generative AI to help me create or revise my résumé”. The scale has demonstrated acceptable reliability in prior Chinese university samples.

#### 3.2.2 Critical thinking

The critical-thinking outcome was operationalised as self-reported critical analytical skills using the eight-item Critical Analytical Skills sub-scale of Hou et al.’s (2022) Chinese Critical Thinking Scale. The sub-scale assesses individuals’ perceived ability to systematically gather and analyse problem-relevant background information and evaluate its veracity. Sample items include “I prefer to follow a structured set of steps when solving problems”, “I often consider the views of my parents and elders when making decisions”, and “I make different decisions in different situations when faced with a problem”. The Chinese Critical Thinking Scale was developed and validated specifically for Chinese university samples and has demonstrated good psychometric properties; higher scores indicate stronger self-reported critical analytical skills.

Following the item-to-construct balance approach (Little et al., 2002), the eight items were aggregated into three parcels for use as indicators of the latent critical analytical skills construct. Specifically, the items were first ranked according to their standardised factor loadings, and relatively higher- and lower-loading items were systematically distributed across the three parcels to balance their relationships with the underlying construct. Parcel scores were calculated as the mean of their constituent items. The resulting three parcels, labelled CT Parcel 1, CT Parcel 2, and CT Parcel 3, were specified as reflective indicators of a single latent critical analytical skills construct. They therefore represent aggregated measurement indicators rather than theoretically distinct dimensions of critical thinking (Little et al., 2002; Matsunaga, 2008).

#### 3.2.3 GenAI feedback literacy

GenAI feedback literacy was assessed using the 39-item Generative AI Student Feedback Literacy Scale (GenAI-SFLS) developed and validated for Chinese college students by Cui et al. (2026). The instrument is organised around four dimensions and nine specific factors: a cognitive dimension (feedback knowledge and feedback appreciation); a behavioural dimension (feedback prompting skills, feedback judgement and enaction of feedback); an affective dimension (emotional recognition, readiness to engage and commitment to change); and an ethical dimension (feedback ethics). The four-dimension architecture extends Carless and Boud’s (2018) general feedback-literacy framework to AI-mediated environments, where prompt engineering and ethical engagement become central to the construct (Cui et al., 2026; Jin et al., 2025; Zhan and Yan, 2026).

#### 3.2.4 Reflective thinking

Reflective thinking was measured with the Reflective Thinking Scale (RTS) developed by Kember et al. (2000), one of the most widely used instruments in this area; in the present study, the Chinese-context revised version validated by Fu and Hali (2025) for use with students in mainland Chinese universities was adopted. The RTS captures four dimensions on a continuum of reflection: habitual action, understanding, reflection and critical reflection, with habitual action representing the least reflective level and critical reflection the most advanced. A sample item is “I often re-appraise my experience so I can learn from it and improve for my next performance”. Items were rated on a five-point Likert scale (1 = definitely disagree, 5 = definitely agree). The four-factor structure has shown good reliability and construct validity in Chinese university samples (Fu and Hali, 2025; Kember et al., 2000).

### 3.3 Data analysis

The analysis proceeded in two stages. First, we screened the data in SPSS 26.0, examined the discriminant validity of the measurement structure with a series of nested confirmatory factor analyses in AMOS 26.0 (maximum likelihood estimation), and tested for common method bias with Harman’s single-factor test and an unmeasured method factor approach (Podsakoff et al., 2003; Tang and Wen, 2020; Wen et al., 2018). Second, the structural model was estimated in SmartPLS 4.1.1.4 using partial least squares structural equation modelling (PLS-SEM). PLS-SEM was selected for three reasons. First, it is well suited to estimating complex structural models with multiple mediators and moderators and to predictive rather than purely confirmatory aims (Hair et al., 2021; Sarstedt et al., 2025). Second, it accommodates multidimensional constructs efficiently, which is relevant because three of the four constructs are multidimensional and were modelled with their dimension scores as reflective indicators, as described below. Third, PLS-SEM performs well with the kind of moderately non-normal Likert data common in higher-education survey research and has been increasingly recommended for moderated mediation tests in this context (Chin et al., 2003; Hair et al., 2021).

Indicator structure and higher-order specification. All four constructs were modelled reflectively with aggregated indicators: dimension scores for GenAI use, GenAI feedback literacy and reflective thinking, and item parcels for critical thinking. For GenAI use, the four scenario sub-scales of Li et al. (2024) (course learning, research activities, daily life, and further study and job seeking) served as indicators; for GenAI feedback literacy, the four dimensions of the GenAI-SFLS (cognitive, behavioural, affective and ethical); and for reflective thinking, the four RTS sub-scales (habitual action, understanding, reflection and critical reflection). Each dimension score was computed as the mean of its constituent items. A reflective (rather than formative) specification was adopted because, in each instrument, the scale developers conceptualise the dimensions as manifestations of a single underlying capacity or disposition rather than as independent components that jointly define it: the dimensions are expected to co-vary, they share common antecedents, and omitting one would narrow the measurement but not alter the conceptual domain of the construct (Hair et al., 2021; Sarstedt et al., 2019). The strong and homogeneous loadings of the dimension scores and parcels (0.755–0.929; Table 2) and AVE values above 0.60 are consistent with this specification. Rather than a repeated-indicator hierarchical model, each multidimensional construct was therefore specified at a single level, with its dimension scores serving as reflective indicators; this specification is analogous to the use of summated dimension scores in the disjoint two-stage approach to higher-order modelling (Sarstedt et al., 2019), except that the dimension scores were computed as item means rather than estimated as latent variable scores in a first stage. The parcelling of the critical thinking items is described in Section 3.2.2; the parcels are labelled CT Parcel 1 to CT Parcel 3 in Table 2 and Figure 2. The two interaction terms were created with the two-stage approach, in which the standardised latent variable scores of the moderator and of the exogenous construct from the main-effects model are multiplied to form a single-indicator interaction term (Hair et al., 2021).

**Table 2:** Estimates of the measurement model.

Construct	Indicator	Loading	Cronbach’s α	CR	ρA	AVE
GenAI use	Further study and job seeking	0.791	0.807	0.873	0.817	0.632
	Daily life	0.782				
	Research activities	0.841				
	Course learning	0.762				
GenAI feedback literacy	Ethical dimension	0.831	0.894	0.927	0.897	0.761
	Affective dimension	0.862				
	Behavioural dimension	0.929				
	Cognitive dimension	0.864				
Critical thinking	CT Parcel 1	0.877	0.761	0.864	0.769	0.680
	CT Parcel 2	0.836				
	CT Parcel 3	0.755				
Reflective thinking	Habitual action	0.875	0.886	0.922	0.889	0.747
	Reflection	0.882				
	Critical reflection	0.851				
	Understanding	0.847				

**Figure 2:**
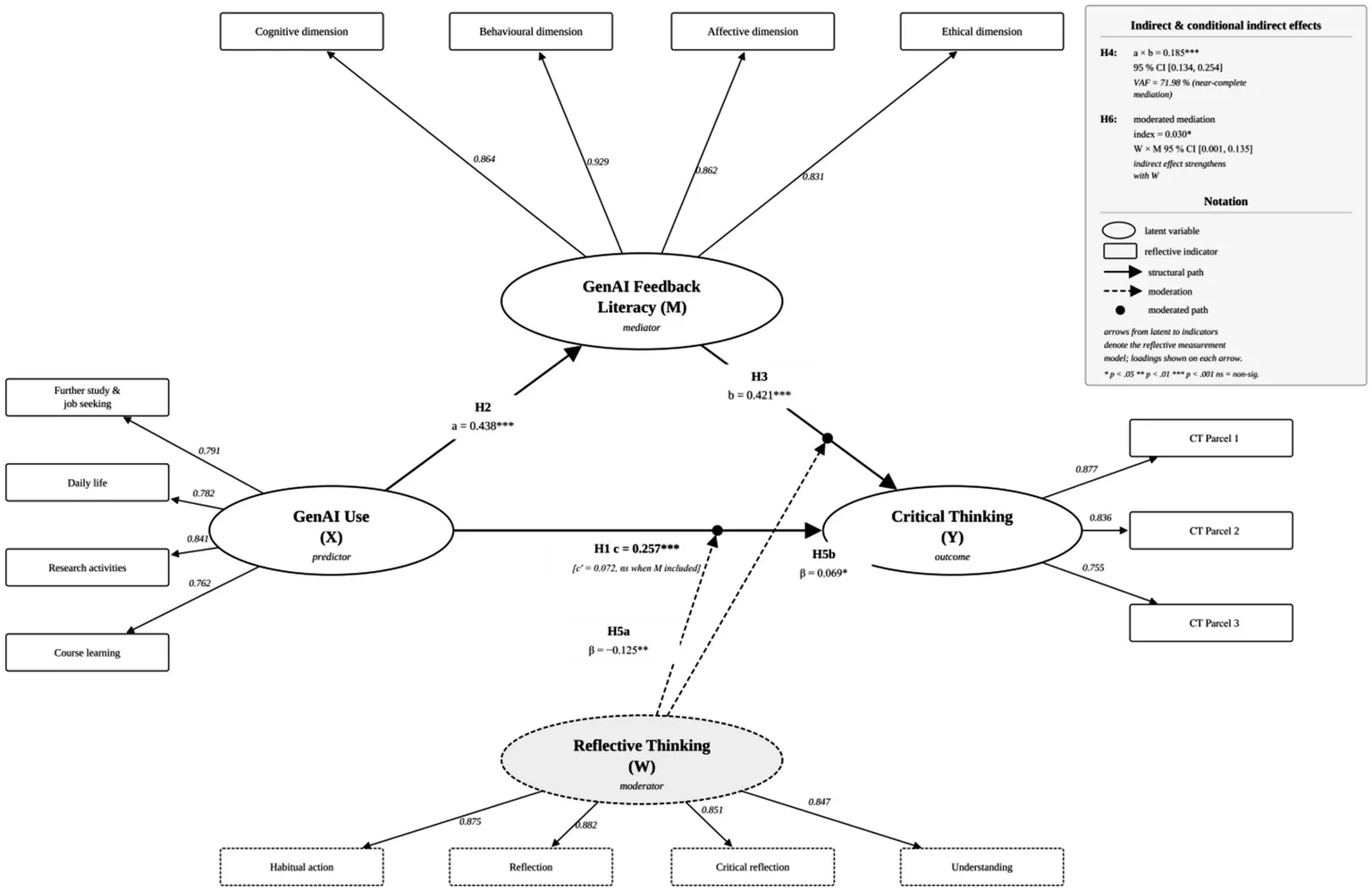
The estimated moderated mediation model with reflective measurement and structural components. Ellipses denote latent variables; rectangles denote observed indicators. Arrows from each latent variable to its indicators specify the reflective measurement model, and indicator loadings are reported in italics on the corresponding arrow. Standardised path coefficients for the structural relationships are shown on the between-construct arrows (5,000 bias-corrected bootstrap resamples). The *c*-coefficient is the total effect of GenAI use on critical thinking; *c*′ is the corresponding direct effect when GenAI feedback literacy is included as a mediator. Filled dots mark paths whose effect on critical thinking is moderated by reflective thinking. ∗*p* < 0.05, ∗∗*p* < 0.01, ∗∗∗*p* < 0.001; *ns* = non-significant.

Indicator reliability, internal consistency, convergent validity and discriminant validity of the measurement model were inspected via factor loadings, Cronbach’s α, composite reliability (CR), the reliability coefficient ρA, average variance extracted (AVE), outer VIF values, the Fornell–Larcker criterion and the heterotrait–monotrait ratio (HTMT). Path coefficients and their statistical significance were estimated with 5,000 bias-corrected bootstrap resamples (Chin, 2009). The mediating effect of GenAI feedback literacy was tested with bias-corrected bootstrap confidence intervals and the variance accounted for (VAF) index (Hair et al., 2014). The moderated mediation model was estimated by adding two interaction terms (Reflective Thinking × GenAI Use; Reflective Thinking × GenAI Feedback Literacy), generated with the two-stage approach described above. The interactions were probed with simple-slope analyses at ± 1 SD of the moderator and with bias-corrected bootstrap confidence intervals for the conditional indirect effects, and moderated mediation was formally tested with the index of moderated mediation (Hayes, 2015), the product of the first-stage path and the second-stage interaction coefficient; because the first-stage path is uniformly positive across bootstrap samples, inference on the index rests on the bias-corrected bootstrap confidence interval of the interaction coefficient. This is in line with current best practise for moderated mediation in PLS-SEM (Hair et al., 2021; Sarstedt et al., 2025). The structural model was further assessed for collinearity (inner VIF), explanatory power (R^2^), effect sizes (f^2^), predictive relevance (Q^2^predict, obtained with the PLSpredict procedure) and overall fit (SRMR), following Hair et al. (2021).

## 4 Results

### 4.1 Common method bias and discriminant validity of the measurement structure

To evaluate the discriminant validity of the four study constructs, a series of nested confirmatory factor analyses was conducted in AMOS 26.0 with maximum likelihood estimation (Table 3). The four-factor model best represented the underlying factor structure (χ^2^/df = 2.189; CFI = 0.731; TLI = 0.723; IFI = 0.732; RMSEA = 0.053), with all factor loadings statistically significant. Although the incremental fit indices (CFI, TLI and IFI) fall below conventional thresholds (a well-documented consequence of model size: incremental fit indices such as CFI and TLI decline as the number of indicators per factor increases, even when RMSEA remains acceptable, and the present item-level model comprises 80 indicators, 39 of them from the GenAI-SFLS; Kenny and McCoach, 2003; Shi et al., 2019), the four-factor model fit was clearly superior to the three-, two- and single-factor alternatives, indicating adequate factorial differentiation amongst GenAI use, GenAI feedback literacy, critical thinking and reflective thinking.

**Table 3:** Confirmatory factor analyses of the measurement model.

Model	Factor structure	χ^2^/df	CFI	TLI	IFI	RMSEA
1	Five-factor	2.900	0.699	0.688	0.602	0.059
2	Four-factor	2.189	0.731	0.723	0.732	0.053
3	Three-factor	2.407	0.681	0.673	0.673	0.058
4	Two-factor	2.737	0.606	0.596	0.608	0.064
5	Single-factor	3.113	0.521	0.508	0.523	0.071

*N* = 421; maximum likelihood estimation in AMOS 26.0. Model 2 is the hypothesised four-factor model (GenAI use, GenAI feedback literacy, critical thinking, reflective thinking). Model 1 adds an unmeasured common method factor, on which all indicators load, to the four substantive factors. Model 3 combines critical thinking and reflective thinking into one factor; Model 4 additionally combines GenAI feedback literacy with critical thinking and reflective thinking; Model 5 loads all indicators on a single factor.

Common method bias was further checked with Harman’s single-factor test (Podsakoff et al., 2003). Twenty-one factors with eigenvalues greater than 1 were extracted from the unrotated solution; the first factor accounted for only 21.46% of the variance, well below the 40% threshold. In the unmeasured method factor approach (Tang and Wen, 2020), adding a common method factor to the four-factor model (Model 1 in Table 3) did not improve model fit (CFI 0.699 vs. 0.731; TLI 0.688 vs. 0.723), indicating that a common method factor does not account for the covariance amongst the indicators (Wen et al., 2018). Both procedures suggest that common method bias is unlikely to be a serious concern in the present data.

### 4.2 Descriptive statistics and zero-order correlations

Table 1 reports means, standard deviations and zero-order correlations amongst the four study variables. GenAI use, GenAI feedback literacy, critical thinking and reflective thinking were significantly and positively inter-correlated (all *p* < 0.001). The pattern is consistent with the proposed model: GenAI use is most strongly correlated with feedback literacy, and feedback literacy and reflective thinking show the strongest associations with critical thinking, anticipating their roles as mediator and moderator.

### 4.3 Measurement model assessment

Table 2 reports the indicator loadings, internal consistency (Cronbach’s α and composite reliability, CR) and convergent validity (average variance extracted, AVE) for each latent construct. All indicator loadings exceeded 0.70, supporting indicator reliability. Cronbach’s α values exceeded 0.70 (Fornell and Larcker, 1981), CR values exceeded 0.80 and the more precise reliability coefficient ρA ranged from 0.769 to 0.897 (Hair et al., 2021), confirming internal consistency. AVE values exceeded 0.60 (Barclay et al., 1995), indicating adequate convergent validity. The square root of each construct’s AVE exceeded its correlations with the other constructs, satisfying the Fornell–Larcker criterion. Heterotrait–monotrait (HTMT) ratios across all construct pairs (0.309, 0.514, 0.583, 0.315, 0.715 and 0.546) were well below the conservative 0.85 threshold (Henseler et al., 2016), supporting discriminant validity. Outer VIF values for the dimension-level indicators and parcels ranged from 1.340 to 2.670, except for the behavioural dimension of GenAI feedback literacy (VIF = 4.019), which exceeds the ideal value of 3 but remains below the critical value of 5 (Hair et al., 2021); indicator collinearity is therefore not a serious concern.

### 4.4 Direct and total effects

Following Chin’s (2009) recommendation, the structural model was estimated in SmartPLS 4.1.1.4 with 5,000 bootstrap resamples. Path coefficients are reported in Table 4. GenAI use significantly predicted GenAI feedback literacy (*β* = 0.438, *t* = 8.335, *p* < 0.001), supporting H2. The total effect of GenAI use on critical thinking, equivalent to its estimate when modelled without the mediator, was also significant (*β* = 0.257, *t* = 4.864, *p* < 0.001), supporting H1. GenAI feedback literacy significantly predicted critical thinking (*β* = 0.421, *t* = 8.309, *p* < 0.001), supporting H3.

**Table 4:** Direct and total effects amongst latent variables.

Path	β (O)	M	SD	*t*	*p*	BootLLCI	BootULCI
GenAI use → GenAI feedback literacy	0.438	0.444	0.053	8.335	<0.001	0.338	0.546
GenAI use → Critical thinking (total effect, model without mediator)	0.257	0.262	0.053	4.864	<0.001	0.155	0.361
GenAI feedback literacy → Critical thinking	0.421	0.426	0.051	8.309	<0.001	0.324	0.525

β (O) = original-sample path coefficient; M = bootstrap mean; SD = bootstrap standard deviation; BootLLCI and BootULCI = lower and upper limits of the 95% bias-corrected bootstrap confidence interval (5,000 resamples). The first and third rows are from the mediation model; the second row is the total effect from the model without the mediator.

### 4.5 The mediating role of GenAI feedback literacy

When GenAI use and GenAI feedback literacy were entered jointly to predict critical thinking, GenAI feedback literacy retained a significant association with critical thinking (*β* = 0.421, *t* = 8.309, *p* < 0.001), whereas the direct association between GenAI use and critical thinking became non-significant (*β* = 0.072, *t* = 1.209, *p* = 0.227). The estimated indirect effect of GenAI use on critical thinking via GenAI feedback literacy was *β* = 0.185 (bootstrap SD = 0.031, *p* < 0.001), with a 95% bias-corrected bootstrap confidence interval of [0.134, 0.254] that excluded zero (Table 5). The variance accounted for (VAF) was 71.98% (0.185/0.257), indicating that nearly three-quarters of the total association between GenAI use and critical thinking is statistically accounted for by feedback literacy, a pattern consistent with near-complete mediation (Hair et al., 2014). H4 is therefore supported.

**Table 5:** Bootstrapped mediation effects.

Path	β (O)	M	SD	*t*	*p*	BootLLCI	BootULCI
Total effect							
GenAI use → Critical thinking	0.257	0.262	0.053	4.864	<0.001	0.155	0.361
Direct effects							
GenAI use → GenAI feedback literacy	0.438	0.444	0.053	8.335	<0.001	0.338	0.546
GenAI use → Critical thinking	0.072	0.073	0.059	1.209	0.227	−0.046	0.187
GenAI feedback literacy → Critical thinking	0.421	0.426	0.051	8.309	<0.001	0.324	0.525
Indirect effect							
GenAI use → Feedback literacy → Critical thinking	0.185	0.189	0.031	6.003	<0.001	0.134	0.254

β (O) = original-sample path coefficient; M = bootstrap mean; SD = bootstrap standard deviation; BootLLCI and BootULCI = lower and upper limits of the 95% bias-corrected bootstrap confidence interval (5,000 resamples).

### 4.6 Moderated mediation analysis

With reflective thinking entered as a moderator, the moderated mediation model was estimated in PLS-SEM (Table 6). The interaction term Reflective Thinking × GenAI Use significantly predicted critical thinking (*β* = −0.125, *t* = 3.138, *p* = 0.002), indicating that reflective thinking moderates the direct relationship between GenAI use and critical thinking. The interaction term Reflective Thinking × GenAI Feedback Literacy also significantly predicted critical thinking (*β* = 0.069, *t* = 1.986, *p* = 0.047), indicating that reflective thinking moderates the relationship between GenAI feedback literacy and critical thinking. Both interactions therefore receive empirical support, and they have opposite signs, consistent with the dual-route reasoning developed in Section 2.3.

**Table 6:** Moderated mediation path estimates.

Path	β (O)	M	SD	*t*	*p*	BootLLCI	BootULCI
GenAI use → Feedback literacy	0.438	0.445	0.053	8.336	<0.001	0.339	0.546
GenAI use → Critical thinking	0.052	0.048	0.051	1.016	0.310	−0.051	0.153
Reflective thinking → Critical thinking	0.487	0.491	0.049	9.940	<0.001	0.395	0.586
RT × GenAI use → Critical thinking	−0.125	−0.121	0.040	3.138	0.002	−0.197	−0.043
RT × Feedback literacy → Critical thinking	0.069	0.071	0.035	1.986	0.047	0.001	0.135
Feedback literacy → Critical thinking	0.174	0.174	0.051	3.420	0.001	0.068	0.275

β (O) = original-sample path coefficient; M = bootstrap mean; SD = bootstrap standard deviation; BootLLCI and BootULCI = lower and upper limits of the 95% bias-corrected bootstrap confidence interval (5,000 resamples). RT = reflective thinking; interaction terms were formed with the two-stage approach.

Simple-slope analyses were carried out to interpret the interactions. For the moderation of GenAI use → critical thinking, GenAI use significantly predicted critical thinking amongst students with low reflective thinking (*β* = 0.177, *t* = 2.678, *p* = 0.008), but the slope was non-significant amongst students with high reflective thinking (*β* = −0.073, *t* = 1.162, *p* = 0.245). For the moderation of GenAI feedback literacy → critical thinking, feedback literacy significantly predicted critical thinking amongst students with high reflective thinking (*β* = 0.243, *t* = 3.704, *p* < 0.001) and the slope was steeper than that observed amongst students with low reflective thinking (*β* = 0.105, *t* = 1.837, *p* = 0.066), where the slope was non-significant. H5a and H5b are therefore supported. As shown in Figure 3, the slope of GenAI use on critical thinking is significantly positive at low reflective thinking and flattens to non-significance at high reflective thinking, supporting H5a. As shown in Figure 4, the slope of feedback literacy on critical thinking is steepest at high reflective thinking and weakest at low reflective thinking, supporting H5b. Together, these two simple-slope patterns are consistent with the predicted dual-route pattern.

**Figure 3:**
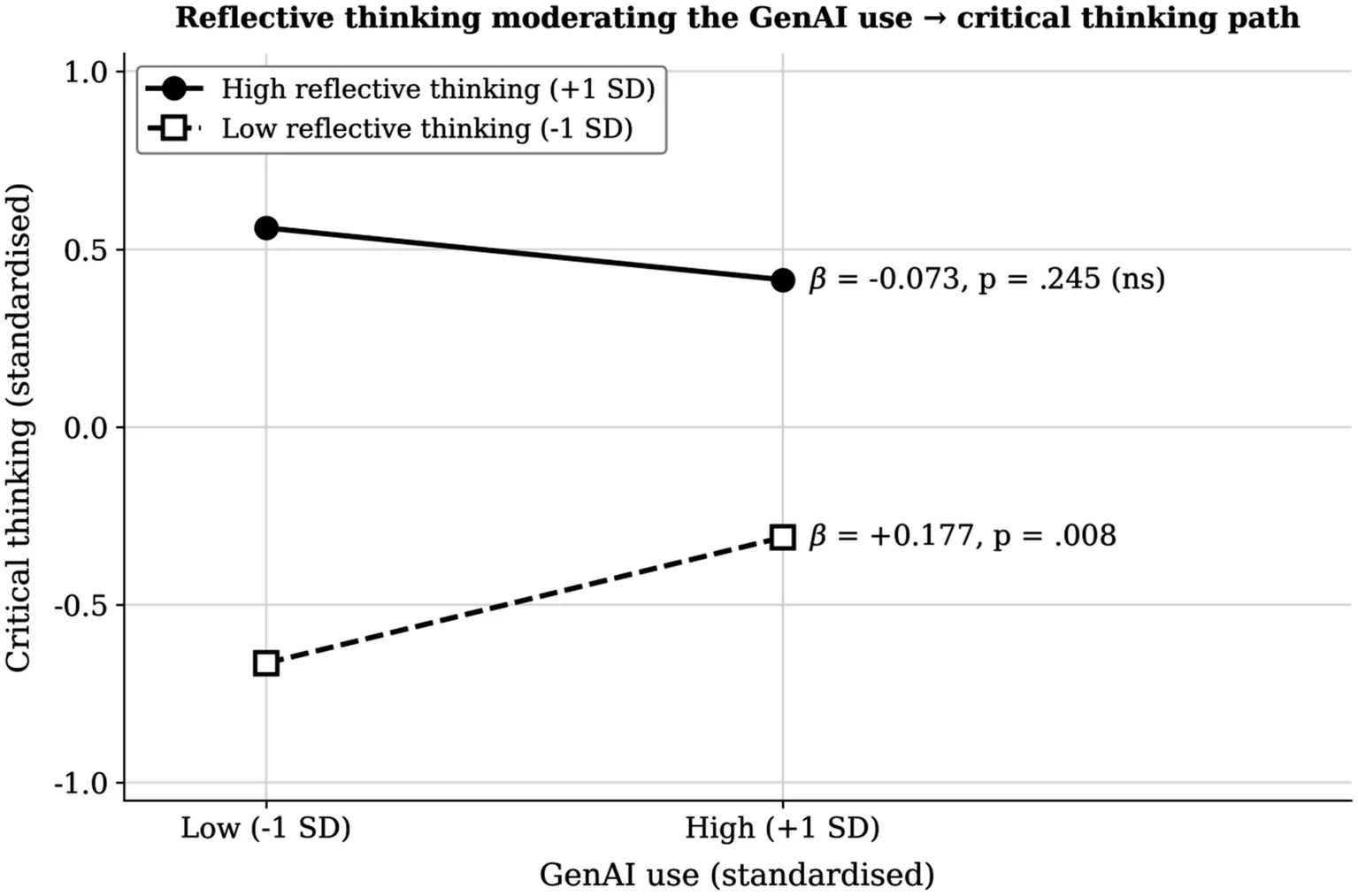
Simple-slope plot of the moderation of GenAI use → critical thinking by reflective thinking. Slopes are estimated at ± 1 *SD* of reflective thinking. At low reflective thinking, GenAI use significantly predicts critical thinking (*β* = +0.177, *p* = 0.008); at high reflective thinking the slope is non-significant (*β* = −0.073, *p* = 0.245). The pattern is consistent with a dual-route interpretation in which the direct path operates primarily for less reflective learners.

**Figure 4:**
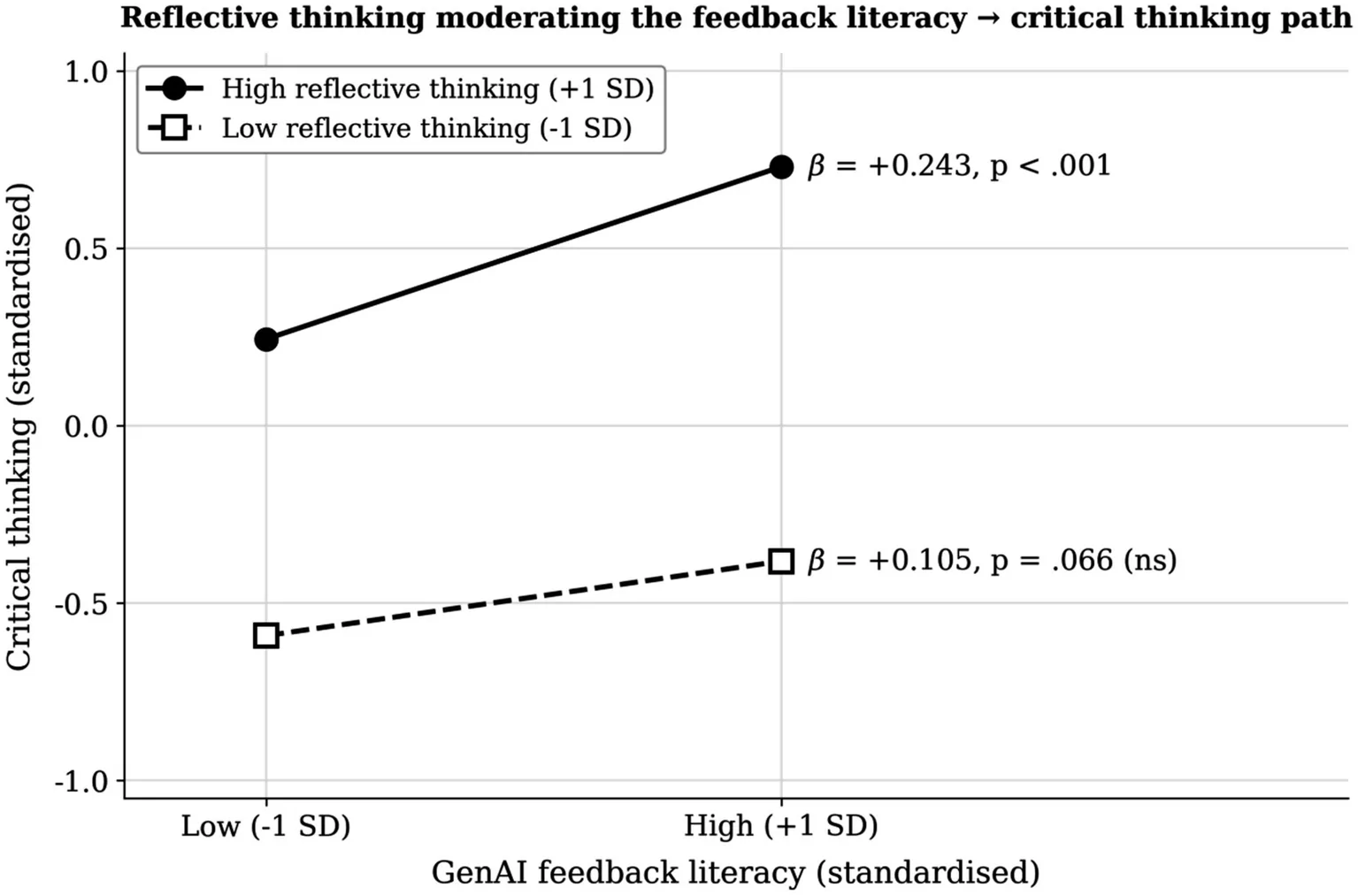
Simple-slope plot of the moderation of GenAI feedback literacy → critical thinking by reflective thinking. At high reflective thinking, feedback literacy strongly predicts critical thinking (*β* = +0.243, *p* < 0.001); at low reflective thinking the slope is shallower and non-significant (*β* = +0.105, *p* = 0.066). The indirect path through feedback literacy is therefore stronger amongst more reflective learners.

To probe the conditional indirect effect, conditional direct and indirect effects were computed at three levels of reflective thinking from the original-sample coefficients, with 95% bias-corrected confidence intervals from the 5,000 bootstrap samples (Table 7). At low reflective thinking (M − 1 SD), the indirect effect of GenAI use on critical thinking through GenAI feedback literacy was not significant (*β* = 0.046, 95% CI [−0.007, 0.100]); the direct effect, however, was significant (*β* = 0.177, 95% CI [0.037, 0.300]). At the mean of reflective thinking, the indirect effect was significant (*β* = 0.076, 95% CI [0.030, 0.128]) whilst the direct effect was not (*β* = 0.052, 95% CI [−0.051, 0.153]). At high reflective thinking (M + 1 SD), the indirect effect remained significant and was the strongest of the three levels (*β* = 0.106, 95% CI [0.047, 0.179]); the direct effect was non-significant (*β* = −0.073, 95% CI [−0.193, 0.053]). Because reflective thinking moderates only the second stage of the indirect path, the index of moderated mediation (Hayes, 2015) is the product of the first-stage path (GenAI use → feedback literacy, a = 0.438) and the coefficient of the Reflective Thinking × Feedback Literacy interaction (b₃ = 0.069): index = a × b₃ = 0.030. The index indicates that the indirect effect increases by 0.030 for each 1 SD increase in reflective thinking; equivalently, the difference between the conditional indirect effects at M + 1 SD and M − 1 SD is 2 × a × b₃ = 0.060 (0.106–0.046). Because the bias-corrected bootstrap confidence interval of a lies entirely above zero ([0.339, 0.546]; Table 6), the sign and significance of the index are determined by b₃, whose bias-corrected bootstrap confidence interval [0.001, 0.135] excludes zero (*p* = 0.047). The index is therefore significantly positive, supporting moderated mediation: as reflective thinking rises, the indirect effect of GenAI use on critical thinking through GenAI feedback literacy strengthens. H6 is supported. Figure 2 visualises the estimated model with reflective measurement indicators and all standardised path coefficients.

**Table 7:** Conditional direct and indirect effects at three levels of reflective thinking.

Path	Reflective thinking	β (O)	*p*	BootLLCI	BootULCI
Direct effect					
GenAI use → Critical thinking	M + 1 SD	−0.073	0.245	−0.193	0.053
GenAI use → Critical thinking	M	0.052	0.310	−0.051	0.153
GenAI use → Critical thinking	M − 1 SD	0.177	0.008	0.037	0.300
Feedback literacy → Critical thinking	M + 1 SD	0.243	< 0.001	0.108	0.372
Feedback literacy → Critical thinking	M	0.174	0.001	0.068	0.275
Feedback literacy → Critical thinking	M − 1 SD	0.105	0.066	−0.014	0.211
Indirect effect					
GenAI use → FL → Critical thinking	M + 1 SD	0.106	0.001	0.047	0.179
GenAI use → FL → Critical thinking	M	0.076	0.002	0.030	0.128
GenAI use → FL → Critical thinking	M − 1 SD	0.046	0.084	−0.007	0.100

Conditional effects are computed from original-sample coefficients (Table 6): direct effect = 0.052 + (−0.125 × RT); feedback literacy → critical thinking = 0.174 + (0.069 × RT); indirect effect = 0.438 × (0.174 + 0.069 × RT), with RT = −1, 0, +1 for M − 1 SD, M and M + 1 SD. FL = GenAI feedback literacy; RT = reflective thinking (standardised).

### 4.7 Structural model assessment

All statistics in this section refer to the full moderated mediation model (Table 6). The structural model was assessed following Hair et al. (2021) before the estimates were interpreted (Table 8). Collinearity amongst the predictors was not a concern: inner VIF values ranged from 1.000 to 1.579, below the conservative threshold of 3. The model explained 19.2% of the variance in GenAI feedback literacy (R^2^ = 0.192) and 39.6% of the variance in critical thinking (R^2^ = 0.396). Effect sizes indicated a medium effect of GenAI use on feedback literacy (f^2^ = 0.238) and of reflective thinking on critical thinking (f^2^ = 0.263), a small effect of feedback literacy on critical thinking (f^2^ = 0.032) and a negligible direct effect of GenAI use on critical thinking (f^2^ = 0.004), consistent with the mediation pattern reported in Section 4.5. The interaction terms had f^2^ values of 0.031 (Reflective Thinking × GenAI Use) and 0.010 (Reflective Thinking × Feedback Literacy). Against Cohen’s general conventions (0.02, 0.15 and 0.35) these are small and below small, respectively; against the benchmarks specific to interaction effects in PLS-SEM (0.005, 0.010 and 0.025 for small, medium and large; Hair et al., 2021), they are large and medium. Because the two-stage interaction terms are products of standardised scores and are not themselves standardised, their f^2^ values are computed from the change in R^2^ when each term is omitted and cannot be recovered from their path coefficients alone. Predictive relevance was assessed with PLSpredict (k = 10 folds, 10 repetitions). Q^2^predict was 0.214 for GenAI feedback literacy and 0.344 for critical thinking, both above zero, indicating that the model has out-of-sample predictive relevance for both endogenous constructs. The standardised root mean square residual (SRMR) of the estimated model was 0.073, below the 0.08 threshold (Henseler et al., 2016).

**Table 8:** Structural model assessment: Collinearity, effect sizes, explanatory power and predictive relevance.

Path/Construct	Inner VIF	*f* ^2^	Effect size	*R* ^2^	Q^2^predict
GenAI use → GenAI feedback literacy	1.000	0.238	Medium	0.192	0.214
GenAI use → Critical thinking	1.241	0.004	Negligible	0.396	0.344
GenAI feedback literacy → Critical thinking	1.579	0.032	Small		
Reflective thinking → Critical thinking	1.498	0.263	Medium		
RT × GenAI use → Critical thinking	1.449	0.031	Small (large for an interaction)		
RT × Feedback literacy → Critical thinking	1.553	0.010	Below small (medium for an interaction)		

All values are from the full moderated mediation model (Table 6). *R*^2^ and *Q*^2^ predict refer to the endogenous construct in each row (GenAI feedback literacy; critical thinking). Effect-size labels follow Cohen’s conventions (0.02 small, 0.15 medium, 0.35 large); labels in parentheses for the interaction terms follow the interaction-specific benchmarks of 0.005, 0.010 and 0.025 (Hair et al., 2021). SRMR (estimated model) = 0.073. RT = reflective thinking.

## 5 Discussion

This study set out to test a moderated mediation model linking generative artificial intelligence use to university students’ self-reported critical thinking, with GenAI feedback literacy as the mechanism and reflective thinking as a boundary condition. Three findings stand out. First, GenAI use was positively related to critical thinking, both directly when modelled alone and through GenAI feedback literacy when both were entered together. Second, the inclusion of feedback literacy reduced the direct effect to non-significance and accounted for nearly three-quarters of the total effect, a pattern consistent with feedback literacy as a principal mechanism. Third, the model was conditional on reflective thinking: at low reflective thinking the link was direct and the indirect path was not significant, whereas at high reflective thinking the indirect path through feedback literacy strengthened and the direct path disappeared. Read together, these results are consistent with a dual-route interpretation of the inconsistent existing evidence on GenAI and critical thinking, in which the structure of the association is sensitive to learner disposition. Each finding warrants discussion.

Before turning to each finding, one interpretive boundary should be made explicit. Critical thinking was measured with the critical analytical skills sub-scale of the Chinese Critical Thinking Scale, on which students rate their own habitual analytical behaviour. The outcome therefore represents self-reported critical analytical skills, not critical-thinking performance demonstrated on a task, a test or an expert-rated piece of work. Self-reports of this kind capture students’ perceptions of how they approach problems rather than the observed quality of their reasoning, and they share a method with the self-reported predictors. Accordingly, where we write that GenAI use, feedback literacy or reflective thinking “predicts” critical thinking, the claim is that these variables are associated with higher self-rated analytical skill in a cross-sectional model, not that they produce better critical-thinking performance. This boundary applies to every finding discussed below.

### 5.1 The link between GenAI use and critical thinking

The positive total association between GenAI use and critical thinking (*β* = 0.257) supports H1 and is consistent with a growing body of evidence that GenAI can scaffold higher-order cognition rather than simply substitute for it (Balart et al., 2026; Hwang et al., 2025; Vargas-Murillo et al., 2026; Wu et al., 2026). The result is consistent with the extended-mind argument that external resources can become functional components of the cognitive system when they participate substantively in information processing (Clark and Chalmers, 1998), and with cognitive load theory’s expectation that absorbing routine sub-tasks frees working-memory resources for analysis and inference (Evans et al., 2024; Sweller, 2024). In the present sample, students who reported higher GenAI use across course learning, research, daily life and career-preparation tasks also reported stronger critical analytical capacity.

At the same time, the direct effect became non-significant once GenAI feedback literacy entered the model. This pattern is congruent with concerns about cognitive offloading and “metacognitive laziness” (Fan et al., 2025; Gerlich, 2025; Risko and Gilbert, 2016; Tian and Zhang, 2025; Zhai et al., 2024). Self-reported GenAI use was not, on its own, associated with higher critical analytical skills once feedback literacy was taken into account; what appears to matter is what learners do with the content the model produces. This dual reading—a positive total effect coexisting with a near-disappearing direct effect—provides a tractable framework for resolving the apparent contradictions in recent reviews (Balart et al., 2026; Vargas-Murillo et al., 2026): bivariate or aggregate analyses detect a positive average association, but they obscure the process that may underlie it. Studies that report only zero-order correlations between AI use and critical thinking therefore risk under-explaining the relationship.

### 5.2 The mediating role of GenAI feedback literacy

Support for H2, H3 and H4 places GenAI feedback literacy at the centre of the model. GenAI use predicted feedback literacy (*β* = 0.438), feedback literacy predicted critical thinking (*β* = 0.421), and the indirect effect was substantial (*β* = 0.185; VAF = 71.98%). This finding extends Carless and Boud’s (2018) feedback-literacy framework to the GenAI context and complements the recent emphasis on AI-specific feedback engagement (Jin et al., 2025; Liu and Deris, 2025; Zhan and Yan, 2026; Zhan et al., 2025; Zhang et al., 2026). Three implications are worth highlighting.

First, the result points to a candidate cognitive mechanism connecting technology use to higher-order thinking, subject to the design caveats noted above. The technology itself does not determine cognitive outcomes; effects are channelled through the cognitive processes that interaction elicits (Yan et al., 2024). What GenAI provides, by virtue of its multi-turn, prompt-and-respond architecture, is an unusually high-frequency feedback environment. Whether learners use that environment to strengthen analysis depends on whether they can comprehend, judge and enact the feedback they receive. Feedback literacy, in this sense, may be the converter that turns interaction frequency into cognitive depth.

Second, the four dimensions of the GenAI-SFLS suggest that domain-specific feedback literacy may differ from generic feedback literacy in important ways. The behavioural dimension includes feedback-prompting skills—an explicitly co-constructive capacity that is unique to AI-mediated feedback—whilst the ethical dimension recognises that critical engagement with GenAI requires judgement about provenance, accuracy and appropriate use (Knoth et al., 2024; Tang et al., 2025; Walton et al., 2026). The strong loading of the behavioural dimension (0.929) underscores the centrality of active prompting and enactment in GenAI feedback literacy. As GenAI agents become more multimodal and tool-augmented, the construct may need to expand further to cover, for example, evaluation of AI-generated visualisations and tool outputs.

Third, the near-disappearance of the direct effect once feedback literacy is added has implications for intervention, provided the mediational ordering is confirmed in longitudinal or experimental work. If it is, encouraging students to use GenAI more without scaffolding their feedback engagement would be unlikely to produce robust cognitive returns (Tepgec et al., 2025). The result supports calls to treat AI feedback literacy as an explicit pedagogical target rather than an emergent by-product of access (Jin et al., 2025; Zhan et al., 2025).

### 5.3 The moderating role of reflective thinking and a dual-route interpretation

The model exhibited a two-pathway structure conditional on reflective thinking. At low reflective thinking (M − 1 SD), GenAI use was directly associated with critical thinking (*β* = 0.177, *p* = 0.008) but the indirect effect was non-significant; at high reflective thinking (M + 1 SD), the direct association disappeared whilst the indirect effect through feedback literacy was strongest (*β* = 0.106, *p* = 0.001). The interaction between reflective thinking and GenAI use on critical thinking was negative (*β* = −0.125), and the interaction between reflective thinking and feedback literacy was positive (*β* = 0.069). The index of moderated mediation was positive and, because the first-stage path is uniformly positive across bootstrap samples, its significance follows from the interaction coefficient, whose confidence interval excluded zero; H5a, H5b and H6 are therefore supported.

These patterns are consistent with a dual-route interpretation, which we advance as a theoretically informed reading of the results rather than as a demonstrated cognitive mechanism. On this reading, less reflective learners show a small, direct association between GenAI use and critical analytical skills that does not pass through systematic feedback engagement; this would be consistent with the modest scaffolding that even relatively passive use can provide, such as organisation, examples and paraphrase (Tian and Zhang, 2025). More reflective learners, by contrast, may treat GenAI outputs as material to be inspected, compared and revised, prompting repeatedly, judging the quality of responses and reworking their own reasoning in light of them; on this account, feedback literacy is the pathway through which their GenAI use relates to critical analytical skills (Chan and Lee, 2021; Walton et al., 2026; Xiao and Liu, 2025). The negative interaction on the direct path does not indicate that GenAI “hurts” critical thinking amongst highly reflective students; statistically, it is consistent with a redistribution of the association into the feedback-literacy pathway, although the model does not test this redistribution directly. We stress that the present study did not observe processing depth directly: no measure of shallow versus deep engagement, and no behavioural trace of how students actually interacted with GenAI, was collected. The terms “shallow” and “deep” therefore describe the theoretical mechanism with which the moderation pattern is consistent, not a mechanism the data demonstrate.

Read in this cautious way, the dual-route interpretation still helps to reconcile the apparently contradictory empirical literature. The cognitive-offloading view may describe less reflective learners, whose GenAI use is, on this interpretation, less tied to systematic feedback engagement (Gerlich, 2025; Tian and Zhang, 2025; Zhai et al., 2024). The cognitive-scaffolding view may describe more reflective learners, whose GenAI use is more strongly associated with an iterative loop of comprehension, evaluation and revision (Hwang et al., 2025; Nasr et al., 2025; Raitskaya and Tikhonova, 2025). The pattern is also compatible with Fan et al.’s (2025) experimental finding that AI-supported learners performed comparably to expert-supported learners on essay outcomes but exhibited fewer self-regulatory and metacognitive moves: similar surface-level performance may be reached via routes of different cognitive depth. Whether only one of those routes builds critical thinking durably is a question for longitudinal and process-tracing designs, not one the present data can answer. The same technology, on this reading, can be a substitute or a partner depending on the metacognitive stance the learner brings to it.

### 5.4 Theoretical implications

The study contributes to the literature in three theoretically substantive ways. First, by integrating extended-mind, cognitive-load and feedback-literacy frameworks, it offers a process-level account of how GenAI use may relate to critical thinking, moving beyond the bivariate “GenAI helps/hurts” debate to a moderated mediation specification consistent with calls for mechanism-focused research in educational technology (Yan et al., 2024). Second, by identifying GenAI feedback literacy as a statistically strong mediator in a cross-sectional model, with VAF approaching the conventional threshold for full mediation, it positions feedback literacy as a plausible principal cognitive interface between AI exposure and higher-order thinking, and extends Carless and Boud’s (2018) framework to AI-mediated environments (Jin et al., 2025; Nieminen and Carless, 2023; Zhan and Yan, 2026). Third, by modelling reflective thinking as a moderator of both the direct and indirect paths, the study identifies a metacognitive boundary condition that may help explain why studies that pool learners produce inconsistent estimates. The dual-route interpretation—a predominantly direct path at low reflectivity and an indirect, feedback-literacy-driven path at high reflectivity—offers a parsimonious and testable frame for future research and a way of reading findings that have so far appeared contradictory.

### 5.5 Practical and pedagogical implications

The findings suggest implications for higher-education policy and practise, which we offer as hypotheses for intervention research rather than as established prescriptions, given the cross-sectional, self-report design. We highlight four. First, GenAI integration policies that focus on usage volume or access alone are unlikely, on the present evidence, to be sufficient for gains in critical thinking. Universities should pair access with explicit feedback-literacy curricula that teach students to comprehend, judge and act on AI-generated feedback, including through prompt-engineering instruction, source-checking exercises and structured comparison of multiple AI outputs (Knoth et al., 2024; Liu and Deris, 2025). Second, instructional design should privilege tasks that require iterative engagement with GenAI rather than one-shot retrieval—for example, multi-stage assignments in which students draft, prompt for feedback, evaluate the response, revise their own work and document the reasoning behind their revisions. Such designs map directly onto the comprehension–evaluation–enaction structure of feedback literacy found to mediate the association with critical thinking in this study, and discourage the metacognitive shortcuts that can occur when AI is used as a one-shot answer machine (Fan et al., 2025).

Third, because the indirect path strengthens with reflective thinking, institutions should embed reflective practises—learning journals, structured debriefs, metacognitive prompts and explicit “think-before-AI” protocols—into AI-augmented coursework, especially in the early years when students are still establishing habits of AI use (Merkebu et al., 2024; Xiao and Liu, 2025). Fourth, the ethical dimension of GenAI feedback literacy deserves explicit curricular attention; teaching students to interrogate GenAI outputs for accuracy, bias, hallucination and appropriate attribution converts ethical engagement into a cognitive resource rather than a compliance issue (Walton et al., 2026). Taken together, these implications imply a reorientation of AI-in-education policy from access and adoption metrics towards measurable feedback-literacy and reflection outcomes, consistent with recent calls in the field (Yan et al., 2024; Zhan et al., 2025).

### 5.6 Limitations and directions for future research

Several limitations bound the study’s claims. First, the cross-sectional design precludes causal inference. All variables were measured concurrently, so the mediation and moderated-mediation estimates describe patterns of association that are consistent with the hypothesised ordering but cannot establish it; reverse or reciprocal orderings (for example, students with stronger analytical skills making heavier or more sophisticated use of GenAI, or feedback literacy shaping use rather than the reverse) are equally compatible with the data. Although PLS-SEM with bootstrapped indirect effects and theoretically motivated path direction is well suited to estimating complex models, the findings should be replicated using longitudinal panel data or randomised intervention designs that track how GenAI use, feedback literacy and reflective thinking co-evolve over time. Second, the sample is drawn from Chinese universities and is skewed female (69.6%); cross-cultural and gender-balanced replications are needed to assess generalisability across educational systems with different assessment cultures and AI-policy environments. Third, all measures are self-reported, and the outcome in particular represents self-reported critical analytical skills rather than demonstrated critical-thinking performance. Although the study’s common-method-bias diagnostics did not flag a serious problem, behavioural and performance-based measures of critical thinking and feedback engagement (e.g., analyses of think-aloud protocols, log data from AI interactions, expert ratings of student work) would strengthen inference (Fan et al., 2025). Fourth, critical thinking was operationalised through the analytical-skills sub-scale of a Chinese instrument; future studies could examine the model with broader operationalisations covering inference, evaluation, explanation and self-regulation (Facione, 2011) and across disciplines, since GenAI’s affordances and risks may differ between, say, the humanities and STEM. Relatedly, the dual-route interpretation in this study rests on the pattern of moderation, not on direct observation of cognitive processing; the study did not measure whether students engaged with GenAI outputs shallowly or deeply, and process-tracing methods such as interaction logs, think-aloud protocols or eye-tracking are needed to test the proposed routes directly. Fifth, the incremental fit indices of the item-level measurement model fell below conventional thresholds, as is common with long instruments such as the 39-item GenAI-SFLS (Kenny and McCoach, 2003; Shi et al., 2019); future work could refine the GenAI-SFLS for shorter administration without losing dimensional coverage. Finally, GenAI tools are evolving rapidly, and the empirical model captures a snapshot of usage patterns at a given moment; tracking the moderated mediation structure across model generations and modalities (text, voice, multimodal agents) is an important agenda for the field.

## 6 Conclusion

Generative AI is rapidly becoming part of the cognitive infrastructure of higher education, and a settled empirical answer to whether its use is associated with stronger or weaker critical thinking has remained elusive. The present study reframes the question. In a cross-sectional sample of Chinese undergraduates, GenAI use was positively associated with self-reported critical analytical skills, but the association was largely indirect: it was carried by students’ capacity to interpret, judge and act on AI-generated feedback, and its structure depended on students’ reflective stance. At low reflective thinking the association was direct and modest; at high reflective thinking it ran through feedback literacy, yielding a stronger indirect association. If these associations reflect the hypothesised causal ordering, a question that longitudinal and experimental designs must settle, the cognitive dividend of GenAI is unlikely to come from access alone, and universities that pair AI integration with explicit feedback-literacy training and reflective pedagogical practises may be better placed to convert ubiquitous AI use into the analytical capacity that higher education is meant to cultivate. The moderated mediation model presented here offers a tractable framework for the next wave of empirical work on GenAI and higher-order cognition.

## Data Availability

The raw data supporting the conclusions of this article will be made available by the authors, without undue reservation.
